# Right ventricular function indices and platelet parameters for early prediction value of bronchopulmonary dysplasia: a retrospective study

**DOI:** 10.1186/s12887-024-04868-y

**Published:** 2024-06-12

**Authors:** Tianzi Li, Bei Xia, Suixin Liang, Qiancheng He, Shuangshuang Zhang, Xiaoyi Chen, Na Xu

**Affiliations:** 1 Department of Ultrasound, Shenzhen Children’s Hospital of China Medical University, Shenzhen, Guangdong China; 2 Department of neonatology, Shenzhen Pediatrics Institute of Shantou University Medical College, Shenzhen, Guangdong China; 3 Department of Ultrasound, Shenzhen Pediatrics Institute of Shantou University Medical College, Shenzhen, Guangdong, China

**Keywords:** Bronchopulmonary dysplasia, Echocardiography, Platelet parameters, Premature infants

## Abstract

**Background:**

To examine the value of early echocardiographic indices for the right ventricular function combined with platelet(PLT) parameters for predicting bronchopulmonary dysplasia (BPD) in preterm infants.

**Methods:**

This retrospective study included infants with gestational age (GA) below 32 weeks, who were admitted to the neonatal intensive care unit(NICU). The detection rate of tricuspid regurgitation jet velocity (TRVJ), ventricular septal flattening, pulmonary artery widening, right ventricular dilation, and right atrial enlargement on the 7th day of life (DOL 7) were compared between BPD and non-BPD infants. Echocardiographic indices of the right ventricular function including tricuspid annular plane systolic excursion (TAPSE) and right ventricular index of myocardial performance (RIMP) were measured on 1 day of life (DOL 1)、on DOL 7 and on 14 day of life (DOL 14) respectively. The PLT parameters including the PLT count, mean platelet volume (MPV), platelet hematocrit (PCT) level, and platelet distribution width (PDW) were measured on the DOL 1,DOL 7, and DOL 14. Multivariate logistic regression was used to analyze the relationship between these parameters and BPD. Receiver operating characteristic curve analysis was performed to assess the predictive value of the right ventricular function indices and PLT parameters for BPD.

**Results:**

A total of 220 preterm infants were included in this study, and of these, 85 infants developed BPD among them. The RIMP of the BPD group on DOL 14 was higher than that of the non-BPD group (*P* < 0.05). The TAPSE of the BPD group on DOL 14 was lower than that of the non-BPD group (*P* < 0.05). The PLT count of the BPD group on DOL 1 was lower than that of the non-BPD group (*P* < 0.05), and the MPV of the BPD group on DOL 1 was higher than that of the non-BPD group (*P* < 0.05). Using multivariate logistic regression, GA、invasive mechanical ventilation duration ≥ 7 days、 PLT、 MPV、 TAPSE and RIMP were found to be independent risk factors for BPD. The area under the receiver operating characteristic curve was 0.846 (95CI: 0.794∼0.899), which improved when using right ventricular function indices combined with platelet parameters.

**Conclusion:**

TAPSE and RIMP combined with PLT count and MPV can help identify preterm infants at an increased risk of developing BPD.

## Introduction

Bronchopulmonary dysplasia (BPD) is among the most common respiratory diseases in premature infants. Recent advancements in respiratory support strategies, and the use of prenatal steroid hormones and pulmonary surfactants, have significantly improved the survival rate of premature infants. Consequently, the number of preterm infants with BPD cases is gradually increasing [1, 2]. BPD not only leads to severe respiratory dysfunction but also results in other complications such as neurological disorders [3] and malnutrition, with poor long-term prognosis. In addition to alveolar development arrest and pulmonary microcirculation disorders, the pulmonary vascular system in BPD also undergoes hypertensive structural remodeling in BPD, leading to the occurrence of pulmonary arterial hypertension [4, 5]. BPD associated with pulmonary hypertension (BPD-PH) contributes significantly to increased morbidity and mortality rates in infants with BPD [6, 7]. Currently, the prevention, treatment and management of BPD has become a challenge in the neonatal intensive care unit (NICU). Identifying infants at an increased risk of BPD using early markers may help deliver timely interventions. However, predicting the risk of BPD development is difficult due to the complex pathophysiology involved.

BPD-PH can be divided into early (10–14 days after birth) and late onset (after 36 weeks postmenstrual age (PMA)) [8]. Early pulmonary vascular disease, assessed through echocardiography on the 7th day of life( DOL 7), is strongly associated with adverse outcomes, including severe BPD, as well as increased risks of PH at 36 weeks PMA and respiratory disease during the first two years of life [9–11]. In general, pulmonary vascular resistance (PVR) is high at birth and then declines during the first weeks of life. However, early or established BPD is characterized by elevated pulmonary resistance. Echocardiographic evaluation of affected infants is a common and important practice. However, the routinely used echocardiographic parameters lack the level of sensitivity required to reliably indicate increased PVR.

A growing body of research has been dedicated to novel echocardiographic parameters such as tricuspid annular plane systolic excursion(TAPSE) and right ventricular index of myocardial performance(RIMP), which may help identify infants at the risk of BPD [12–15]. Several studies [12, 13]have shown that BPD-PH infants have higher RIMP and lower TAPSE. But most of these studies were based on assessments at 36 weeks PMA and beyond. Neumann et al. found that RIMP and birth weight (BW) measured on DOL 7 were independent risk factors for BPD [14]. Mendez-Abad et al. found an association of TAPSE at DOL 14 with the development of BPD [15]. The development of alveolar microvasculature can promote the formation of alveoli structure formation, which has become the focus of BPD-related research. Platelets play an essential role in the formation and development of pulmonary blood vessels. Several studies have shown that peripheral PLT parameters may help evaluate the risk of BPD in preterm infants [16–19]. Echocardiography and PLT parameters are easy to obtain and monitor in the clinical practice. However, these sets of parameters have not been previously investigated simultaneously in preterm infants. This study aimed to investigate the ability of the predictive value of echocardiographic indices and PLT parameters for BPD at 36 weeks PMA.

## Methods

### Study design

This was a retrospective study, conducted by reviewing medical records and data. The study protocol was approved by the institutional research ethics committee of Shenzhen Children’s Hospital (2,022,062).

### Patients

This retrospective study was performed on data from 462 premature infants with a gestational age(GA) of < 32 weeks who were hospitalized between January 2019 and December 2021 in the NICU at Shenzhen Children’s Hospital.

#### The inclusion criteria were as follows

(1)GA at birth < 32 weeks; (2)admitted to hospital within one day after birth; (3)the hospital stay was longer than 28 days; (4)echocardiography was performed on DOL 1、DOL 7、 and DOL 14; (5)platelet tests were performed on DOL 1、DOL 7 and DOL 14.

#### The exclusion criteria were as follows

(1) severe congenital constructional or chromosome malformations, such as congenital heart or lung disease(infants with patent foramen ovale(PFO) and no hemodynamically significant patent ductus arterious(no hsPDA) were excluded), diaphragmatic hernia, genetic metabolic disease, or chromosomal disease; (2) blood exchange treatment or suffering from hematological diseases; (3) neonatal sepsis and neonatal severe asphyxia; (4)severe arrhythmia; (5)poor echocardiography image quality or incomplete clinical medical records.

### Diagnostic criteria

According to the consensus definition of the National Institute of Child Health and Human Development [20], BPD is diagnosed when supplemental oxygen is required for more than 28 days. BPD severity was assessed according to the oxygen concentration required at 36 weeks PMA or at discharge.

**Fig. 1:**
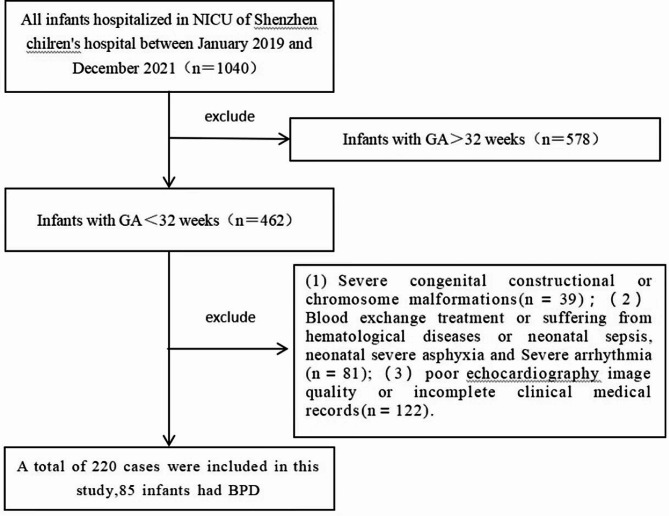
Flowchart of cases selection and analysis. 220 premature infants were enrolled in this study. NICU, neonatal intensive care unit; GA, gestational age; BPD, bronchopulmonary dysplasia

We enrolled 220 premature infants in the study including 85 and 135 infants in the BPD group and non-BPD group respectively (Fig. 1).

### Clinical data collection

Data on the following variables were retrieved from the electronic medical records: GA, birth weight(BW), delivery method, Apgar score at 1 min, sex, multiple births, surfactant treatment, duration of invasive mechanical ventilation, neonatal pneumonia and neonatal respiratory distress syndrome (NRDS), maternal age, antenatal steroid use, antibiotics use, incidence of premature prelabor rupture of membranes, gestational diabetes mellitus and high blood pressure during pregnancy.

### Echocardiographic measurements

Echocardiographic evaluation was performed using a Vivid E9 (GE Health care, Horten, Norway) with an M6S probe. All infants were in a state of quiet sleep cooperation at the time of examination. All images and measurements were obtained from standard views according to the recommendations of the American Society of Echocardiography for chamber quantification [21]. When the two-dimensional image section was stable, clear and standard, dynamic images of three cardiac cycles were stored and the original data were measured again.

All measurements were taken by two senior cardiac sonographers.

#### Traditional ultrasonic indices

The detection rate of tricuspid regurgitation jet velocity (TRJV), ventricular septal flattening, pulmonary artery widening, right ventricular dilation and right atrial enlargement were analyzed on DOL7 in the two groups (Table 2).

**Fig. 2:**
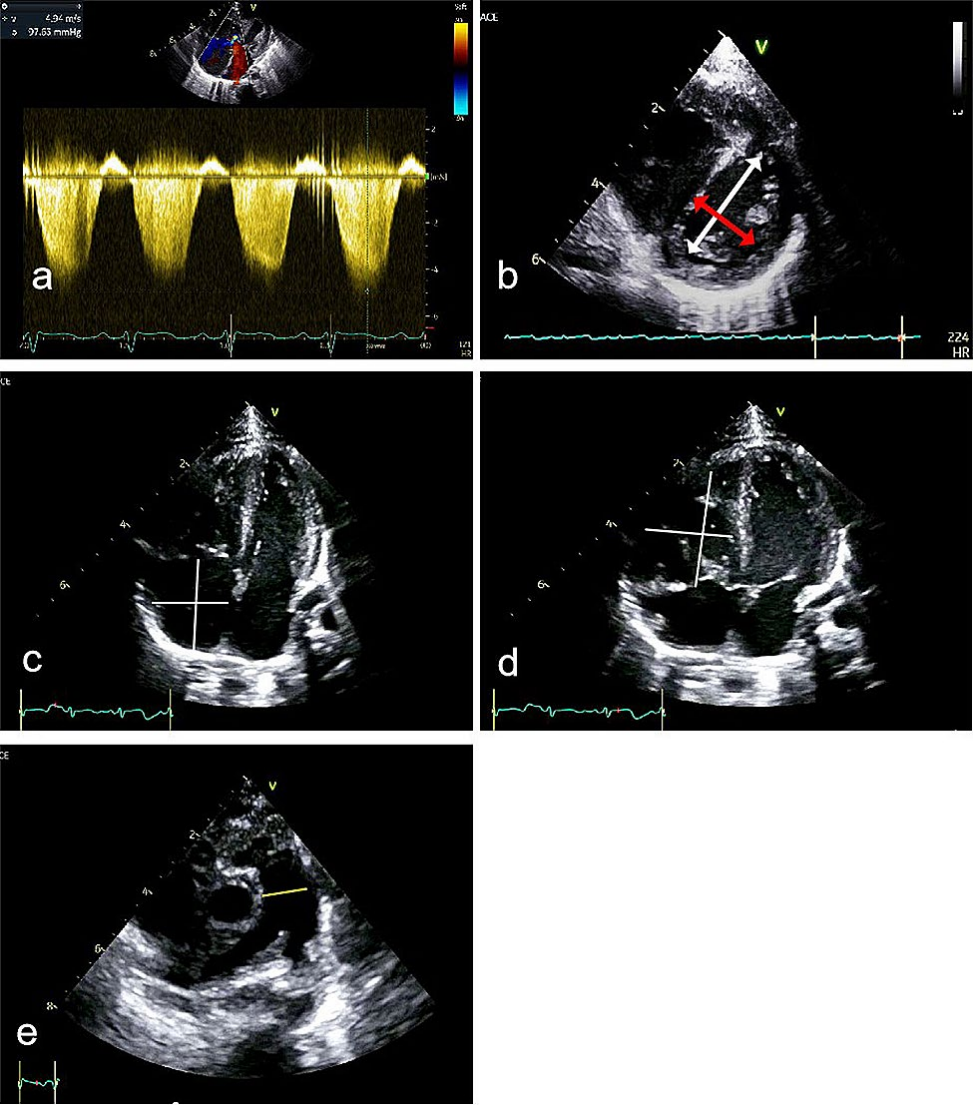
Measurement of the traditional ultrasonic indices. (**a**) tricuspid regurgitation jet velocity was measured using continuous doppler; (**b**) The white line represents “D1” and the red line represents “D2” ,left ventricular eccentricity index(LVEI) was obtained through D1 / D2 calculations; (**c-d**) The inner diameter of right atrium and right ventricle were measured in the four-chamber section of the apex; (**e**) The inner diameter of pulmonary arteries were measured in the short axis of the aorta

①TRJV was measured using continuous doppler. The modified Bernoullik method was used to estimate right ventricular systolic pressure (RVSP), and in the absence of right ventricular outflow tract obstruction, RVSP was equal to pulmonary systolic pressure (PASP): PASP = RVSP = 4TRJV ^2^+RAP (Fig. 2-a).

②Ventricular septal flattening can be quantified using the left ventricular eccentricity index(LVEI). The left ventricular long diameter D1 parallel to the interventricular septum and the left ventricular short diameter D2 perpendicular to the septum were measured on the parastericular short-axis section. LVEI was obtained through D1 / D2 calculations (Fig. 2-b).

③The inner diameter of right atrium and right ventricle were measured in the four-chamber section of the apex (Fig. 2-c and 2-d ) and determines whether dilated according to the normal reference value [22].

④The inner diameter of the main pulmonary artery and left and right pulmonary arteries were measured in the short axis of the aorta (Fig. 2-e) and determines whether dilated according to the normal reference value [22].

#### Right ventricular function indices

We performed transthoracic echocardiography on DOL 1,DOL 7 and DOL 14.

**Fig. 3:**
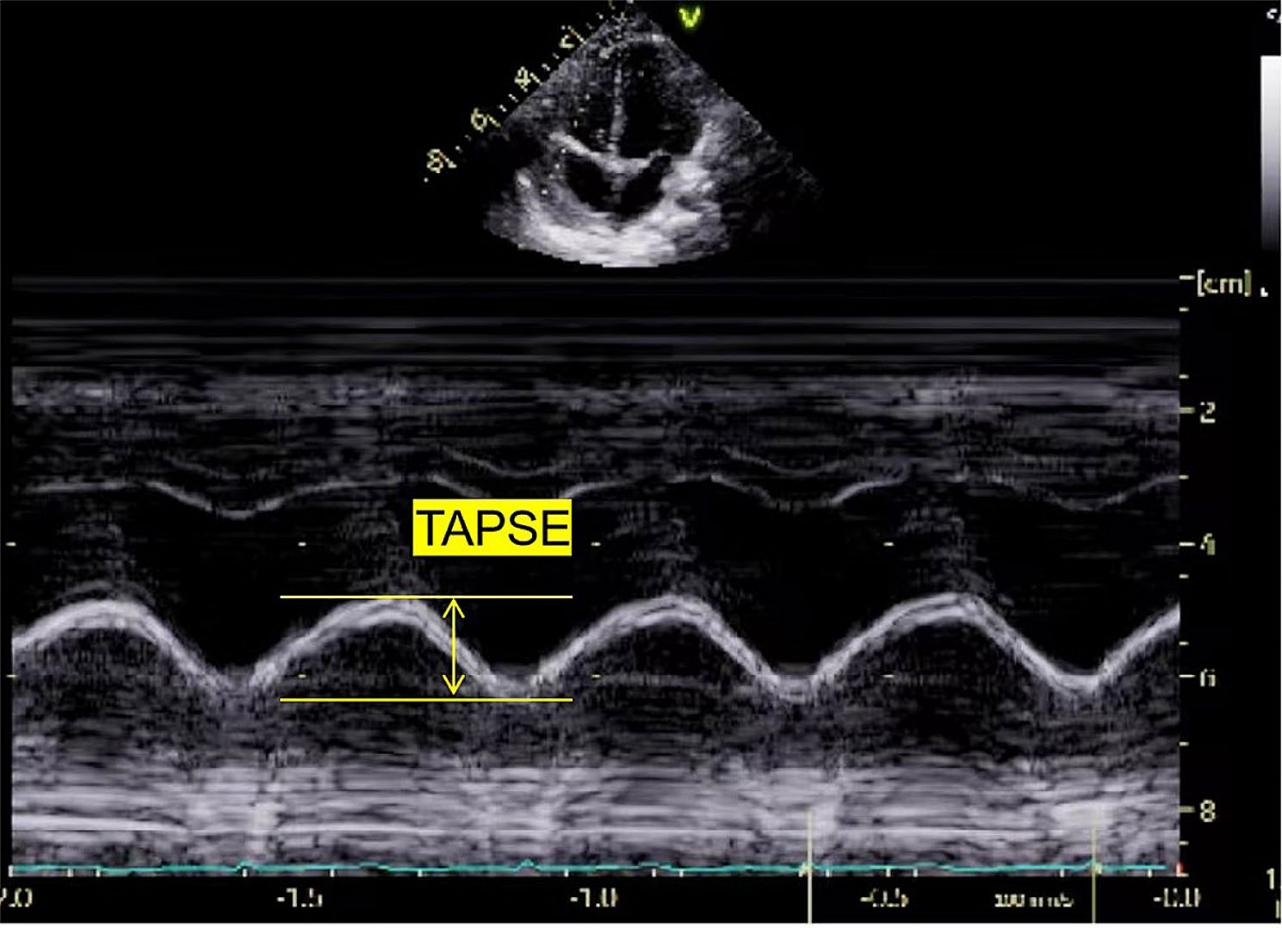
Measurement of TAPSE. The distance between the end-diastolic and end-systolic represents TAPSE

①TAPSE was measured through M-mode from the four-chamber view. M sampling lines are parallel to the direction of movement of the tricuspid annulus of the sidewall. The distance between the end-diastolic and end-systolic represents TAPSE (Fig. 3).

**Fig. 4:**
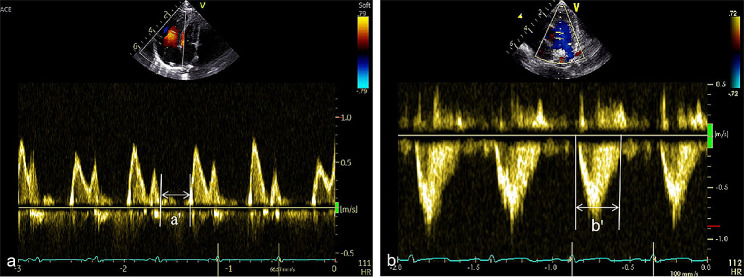
Measurement of RIMP. The “**a'**” interval was the time from tricuspid closing to opening.the “**b'**” interval was measured between onset and cessation of the right ventricle outflow and RIMP was calculated as (a'-b')/b'

②RIMP was measured with conventional pulsed Doppler using the method proposed by Tei et al. Pulsed-Doppler waveforms of the tricuspid inflow were recorded from the parasternal four-chamber view and the “a'’” interval was the time from tricuspid closing to opening. The right ventricular outflow patterns were visualized from the parasternal short-axis view, and the “b'’” interval was measured between onset and cessation of the right ventricle outflow and RIMP was calculated as (a'-b')/b'. To minimize variations in heart rate, mean values were obtained by averaging a minimum of three consecutive cardiac cycles (Fig. 4).

### Measurement of platelet parameters

Platelet parameters were recorded on DOL 1,DOL 7, and DOL 14, including platelet (PLT) count, mean platelet volume (MPV), platelet hematocrit (PCT) level, and platelet distribution width (PDW). The blood cell analyzer SysmexXN350 and the supporting reagent of Micron Hisen were used for parameter detection.

### Statistical analysis

Statistical analysis was performed using SPSS 26.0 software. The normality of continuous variable distribution assumption was tested. Normally distributed variables were presented as the mean ± standard deviation, and non-normally distributed variables were presented as the median and quartile range. Comparisons between the continuous variables were performed using an independent sample t-test or Mann-Whiteny U-test. Categorical variables were presented as the frequency and percentage and were compared using the chi-squared test. The Friedman test was used to investigate the age dependency of TAPSE and RIMP. Multivariate logistic regression was performed to determine the independent risk factors of BPD. Odds ratios (OR) and 95% confidence intervals (CI) were calculated in logistic regression analysis. A receiver operating characteristics (ROC) curve was used to analyze the accuracy of echocardiographic indices of the right ventricular function combined with platelet parameters in predicting BPD. *P*-values of < 0.05 were considered statistically significant.

To ensure the stability of the Logistic regression model, a multicollinearity test was conducted on the variables included in the regression. Blant-Altman analysis was used to test the inter-group and intra-group repeatability of TAPSE and RIMP.

## Results

### Patient characteristics

A total of 220 patients were included in this study, among them, 85 infants had BPD (Table 1). The BPD infants had lower GA, BW than did the non-BPD infants (*P* < 0.05). The infants in the BPD group received more invasive mechanical ventilation for a duration ≥ 7 days and more alveolar surfactant than did those in the non-BPD group (*P* < 0.05). There were no significant differences between the two groups in terms of sex, 1-minute Apgar score ≤ 7,mode of delivery, multiple birth incidence, test-tube baby, neonatal pneumonia, neonatal respiratory distress syndrome (NRDS), maternal age, antenatal steroid use, antibiotics use, incidence of premature prelabor rupture of membranes, gestational diabetes mellitus and high blood pressure during pregnancy. (*P* > 0.05).

**Table 1:** Characteristics of the infants with and without BPD

Variables	BPD (*n*=85)	Non- BPD (*n*=135)	t/X^2^	*p*
GA, weeks	28.0(26.8, 29.2)	29.6(28.4, 31.0)	-6.71	<0.001
BW, grams	1050(900, 1200)	1390(1090, 1520)	-6.02	<0.001
1-min Apgar score ≤ 7(%)	38(44.7%)	46(34.1%)	2.50	0.11
male(%)	45(52.9%)	79(58.5%)	0.66	0.42
Cesarean delivery(%)	38(44.7%)	63(46.7%)	0.08	0.78
Test-tube baby (%)	18(21.2%)	25(18.5%)	0.23	0.63
Multiple birth (%)	28(32.9%)	43(31.8%)	0.03	0.87
Invasive mechanical ventilation duration ≥ 7 days(%)	61(71.8%)	61(45.2%)	14.92	0.00
Neonatal pneumonia(%)	29(34.1%)	40(29.6%)	0.49	0.49
NRDS(%)	55(64.7%)	77(57.0%)	1.28	0.26
Surfactant administration (%)	44(51.7%)	49(36.3%)	5.11	0.02
Maternal age(year)	30(27, 35)	30(27, 32)	-1.05	0.29
Premature rupture of membranes(%)	28(32.9%)	36(26.7%)	1.00	0.32
Prenatal steroid use (%)	36(19.5%)	49(36.3%)	0.81	0.37
Antibiotics use(%)	14(16.5%)	15(11.1%)	1.31	0.25
Gestational diabetes mellitus(%)	13(15.3%)	31(23.0%)	1.96	0.17
High blood pressure during pregnancy(%)	16(18.8%)	17(12.6%)	1.59	0.21

Data were displayed as median(interquartile range) or number(percentage)

*BPD*, bronchopulmonary dysplasia;*GA*, gestational age;*BW*,birth weight;

*NRDS*,neonatal respiratory distress syndrome

### Traditional echocardiographic parameters

There were no significant differences between the two groups in the detection rate of TRJV, ventricular septal flattening, pulmonary artery widening, right ventricular dilation, right atrial enlargement on DOL 7(*P*>0.05), as shown in Table 2.

**Table 2:** Traditional echocardiographic parameters of the infants with and without BPD

Traditional echocardiographic parameters	BPD	Non-BPD	*P*
TRJV(%)	50(58.8%)	69(51.1%)	0.26
ventricular septal flattening(%)	10(11.7%)	9(6.7%)	0.19
pulmonary artery widening(%)	13(15.3%)	13(9.6%)	0.21
right ventricular dilation(%)	11(12.9%)	10(7.4%)	0.17
right atrial enlargement(%)	10(11.7%)	9(6.7%)	0.19

Data were displayed as number(percentage). *TRJV*,tricuspid regurgitation jet velocity

### Comparison of right ventricular function indices

**Fig. 5:**
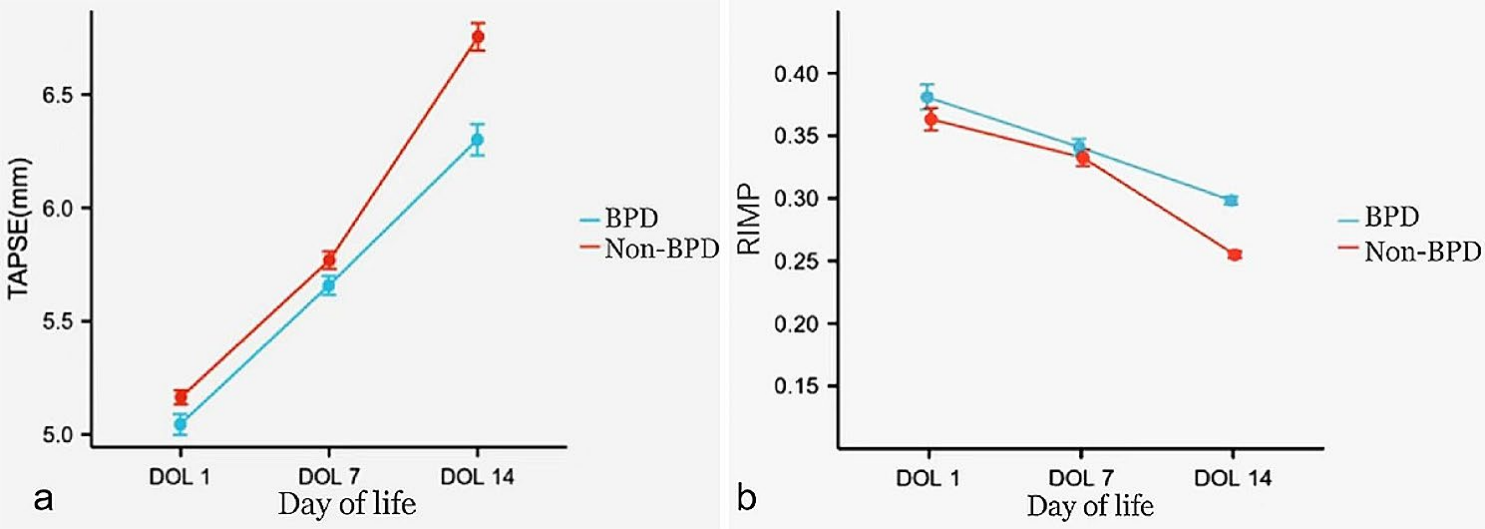
Comparison of trends for TAPSE and RIMP from DOL 1 to DOL 14 between the two groups

The increase in TAPSE and the decrease in RIMP during the first 14 days of life differed significantly between the non-BPD infants and BPD infants (Fig. 5). TAPSE values increased faster from DOL 7 to DOL 14 in infants without BPD than in infants who developed BPD. In infants who did not develop BPD, a rapid increase occurred from DOL 7 to DOL 14, whereas the TAPSE of infants who developed BPD remained low. Between days 1 and 7 the increase in TAPSE was approximately equal in both groups. RIMP values decreased faster from DOL 7 to DOL 14 in infants without BPD than in infants who developed BPD. In infants who did not develop BPD, a rapid decrease occurred from DOL 7 to DOL 14, whereas the RIMP of infants who developed BPD remained high. Between days 1 and 7 the decline in RIMP was approximately equal in both groups. The values of TAPSE were lower, and those of RIMP were higher, in infants with BPD than in those without BPD at DOL 14 (6.40 [5.90–6.90] vs. 6.80[6.10–7.10], *P* < 0.001 ; 0.30[0.29–0.31] vs. 0.26[0.23–0.28], *P* < 0.001 ) (Tables 3 and 4 ).

**Table 3:** Comparison of TAPSE between the two groups at DOL 1,DOL 7, and DOL 14

TAPSE(mm)	BPD	Non-BPD	*P*
DOL 1	5.00(4.70, 5.30)	5.10(5.00, 5.40)	0.12
DOL 7	5.70(5.40, 5.90)	5.70(5.48, 6.00)	0.07
DOL 14	6.40(5.90, 6.90)	6.80(6.10, 7.10)	<0.001
*P*	<0.001	<0.001	

Data were displayed as median(interquartile range).*TAPSE*, tricuspid annular plane systolic excursion

**Table 4:** Comparison of RIMP between the two groups at DOL 1,DOL 7, and DOL 14

RIMP	BPD	Non-BPD	*P*
DOL1	0.38(0.29, 0.46)	0.40(0.26, 0.45)	0.35
DOL7	0.34(0.30, 0.37)	0.32(0.29, 0.39)	0.21
DOL14	0.30(0.29, 0.31)	0.26(0.23, 0.28)	<0.001
*P*	<0.001	<0.001	

Data were displayed as median(interquartile range) .*RIMP*,right ventricular index of myocardial performance

### Comparison of platelet parameters in BPD group and no-BPD group

The PLT count of the BPD group was lower and the MPV was higher on DOL 1 than those in the non-BPD group (both *P* < 0.05). The PCT and PDW values were not comparable in both groups at each time (Table 5).

**Table 5:** Comparison of platelet parameters between BPD group and non-BPD groups

Platelet parameters	DOL 1		t, *P*	DOL 7		t, *P*	DOL 14		t, *P*
	BPD	No-BPD		BPD	No-BPD		BPD	No-BPD	
PLT(×10^9^/L)	207.21 ± 46.26	223.10 ± 48.05	-2.42,0.02	213.19 ± 77.66	234.03 ± 72.57	-2.02,0.05	228.07 ± 84.46	243.25 ± 49.77	-1.50,0.14
MPV(fl.)	10.67 ± 0.88	10.35 ± 0.86	2.54,0.01	11.68 ± 1.00	11.95 ± 1.05	-1.86,0.06	11.58 ± 0.84	11.79 ± 1.00	-1.61,0.11
PCT(%)	0.22 ± 0.06	0.22 ± 0.07	0.14,0.89	0.25 ± 0.08	0.27 ± 0.08	-1.98,0.05	0.32 ± 0.10	0.34 ± 0.11	-1.67,0.10
PDW(fl.)	12.42 ± 2.24	11.99 ± 2.36	1.33,0.18	15.13 ± 3.49	14.55 ± 3.04	1.56,0.12	16.22 ± 2.81	15.76 ± 2.74	1.20,0.23

Data were displayed as mean ± standard deviation.*PLT*, platelet count;*PDW*, platelet distribution width;*PCT*, plateletcrit;*MPV*, mean platelet volume

### Logistic regression analysis of risk factors for BPD

After adjusting some potential confounders including BW, and surfactant administration, multivariate logistic regression analysis revealed that GA (OR = 1.97, 95% CI: 1.057–3.682), invasive mechanical ventilation duration ≥ 7 days (OR = 0.35, 95% CI: 0.171–0.715), PLT on DOL 1 (OR = 1.46, 95% CI: 1.077–1.979), MPV on DOL 1(OR = 1.02, 95% CI: 1.007–1.034), TAPSE on DOL 14 (OR = 1.59, 95% CI: 1.03–2.300) ,and RIMP on DOL 14 (OR = 1.67, 95% CI: 1.242–2.243) were independent risk factors for BPD, as shown in Table 6.

**Table 6:** Logistic regression analysis of risk factors for BPD

	β	SE	Wald	*P*	Exp (β)	95% CI for Exp(β)
GA	0.68	0.32	4.55	0.03	1.97	(1.057∼3.682)
BW	0.11	0.24	0.19	0.67	1.11	(0.690∼1.789)
Invasive mechanical ventilation duration ≥ 7 days	-1.05	0.65	8.29	0.00	0.35	(0.171∼0.715)
Surfactant administration	-0.71	0.36	3.82	0.05	0.49	(0.242∼1.002)
PLT	0.38	0.16	5.96	0.02	1.46	(1.077∼1.979)
MPV	0.02	0.01	8.63	0.00	1.02	(1.007∼1.034)
TAPSE on DOL 14	0.47	0.19	6.17	0.01	1.59	(1.103∼2.300)
RIMP on DOL 14	0.51	0.15	11.54	0.00	1.67	(1.242∼2.243)
constant	-1.49	5.90	0.07	0.80	0.22	

*β*, estimated regression coefficient;*SE*, standard error;*Exp (β)*, exponentiation of estimated regression coefficient;*CI*,confidence intervals

### Predictive value of different parameters

**Fig. 6:**
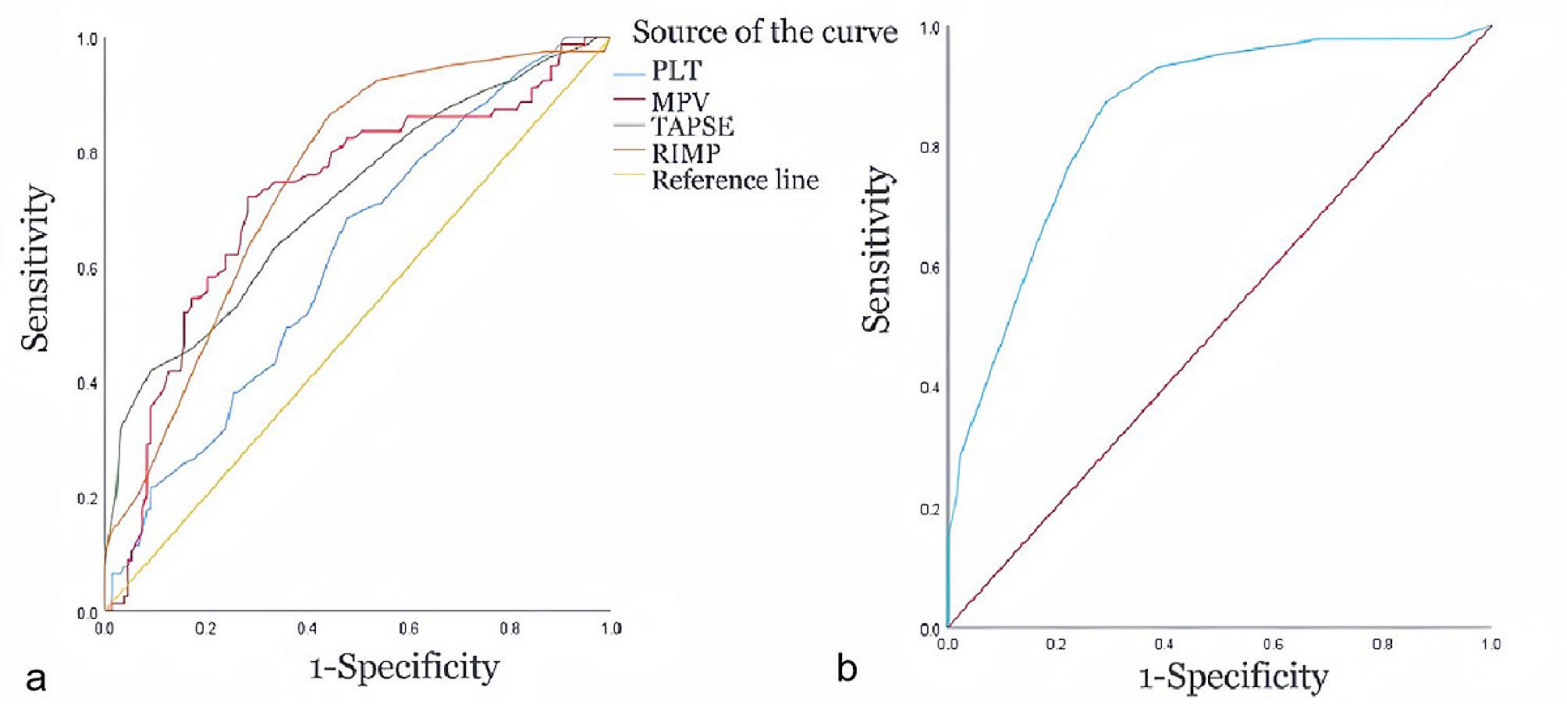
Receiver operating characteristic curve for predicting BPD. ROC curves for predicting BPD with TAPSE, RIMP, PLT and MPV

The ROC curve analysis for the detection of BPD revealed that RIMP and TAPSE showed similar areas under the curve (AUC), with RIMP showing the largest and MPV showing the smallest AUC. With the cutoff value of RIMP at 0.27, the diagnostic accuracy showed a sensitivity of 0.861 and a specificity of 0.709. With the cutoff value of TAPSE at 5.75, the diagnostic accuracy showed a sensitivity of 0.418 and a specificity of 0.910. With the cutoff value of PLT at 197.34, the diagnostic accuracy showed a sensitivity of 0.722 and a specificity of 0.716. With the cutoff value of MPV at 10.25, the diagnostic accuracy showed a sensitivity of 0.684 and a specificity of 0.522. The combination of these parameters helped predict BPD with an AUC of 0.846 (95% CI, 0.794–0.899), as shown in Table 7; Fig. 6.

**Table 7:** Predictive value of different parameters

Parameters	Cut off value	AUC	95%CI	*P*	Sensitivity	Specificity
TAPSE on DOL 14	5.75	0.714	0.641–0.787	<0.001	0.418	0.910
RIMP on DOL 14	0.27	0.749	0.681–0.815	<0.001	0.861	0.709
PLT	197.34	0.721	0.647–0.795	0.005	0.722	0.716
MPV	10.25	0.616	0.539–0.692	<0.001	0.684	0.522
Combined	—	0.846	0.794–0.899	0.000	0.871	0.881

Each parameter corresponds to a cut-off value.*AUC*, areas under the curve

### Repeatability test

Blant-Altman analysis was performed to test the inter-observer and intra- observer agreement of TAPSE and RIMP. The results showed that the reproducibility of the measurement of TAPSE and RIMP was good, as shown in Table 8.

**Table 8:** The inter-observer and intra-observer agreement of TAPSE and RIMP

Parameters	mean ± standard deviation	mean ± standard deviation	deviation	*P*	95% CI
inter-observer agreement					
TAPSE	5.60 ± 0.41	5.60 ± 0.38	0.01	0.75	(-0.03,0.04)
RIMP	0.33 ± 0.12	0.33 ± 0.12	0.00	0.52	(-0.01,0.01)
intra-observer agreement					
TAPSE	5.60 ± 0.41	5.59 ± 0.40	0.00	0.81	(-0.03, 0.03)
RIMP	0.33 ± 0.12	0.33 ± 0.11	0.01	0.24	(-0.03, 0.05)

Data were displayed as mean ± standard deviation

## Discussion

In this study, GA and invasive mechanical ventilation ≥ 7 days were found to be clinically independent risk factors for BPD, which was consistent with previous research [23–25]. The smaller the GA is, the less mature the lung and airway structures are. Therefore, the lung is more likely to be affected by various high-risk factors and suffer damage. For premature infants who require assisted breathing and oxygen support, invasive mechanical ventilation strongly stimulates the respiratory tract, aggravates lung inflammation and promotes lung injury [24].

Echocardiography has been the most commonly used tool in neonatal care department to evaluate pulmonary artery pressure and right heart function in infants with BPD-PH. Mourani et al. [10] found that PH signs (including ventricular septal flattening and right ventricular dilation) observed through echocardiography in premature infants at DOL 7 were associated with BPD. However, early identification of PH remains difficult. Mirza H et al. found that measurable TRJV could only be obtained in about 31–61% of infants with BPD combined with PH or suspected PH [8]. In this study, we found that the detection rate of TRJV measured through echocardiography was only 58.8%. In addition, ventricular septal flattening (11.7%), pulmonary artery widening (15.3%), right ventricular dilatation (12.9%), and right atrial enlargement (11.7%) were low. These signs are more obvious when the right ventricular afterload increases significantly, and therefore is not a sensitive indicator for monitoring PH .

We studied two echocardiographic indices of right ventricular function, namely RIMP and TAPSE in very preterm infants. Both indices are established markers in adults for echocardiographic right heart assessment but their value for the prediction of BPD is not well known. Our results reveal that RIMP values are relatively high at DOL 1 but fall rapidly similar to the drop and confirmed the longitudinal time course of RIMP during the neonatal period as already demonstrated by Murase et al [26]. For newborns, the transition from intrauterine to extrauterine environment is a critical stage, and PVR decreases with the establishment of spontaneous breathing after birth. Two weeks after birth, there was a difference in the trend of decline in RIMP between the two groups. The decreasing trend of RIMP from DOL1 to DOL7 between the two groups was similar, which may be due to the improvement in pulmonary vascular compliance, the decrease in PVR, and the gradual decrease in the need for respiratory support in premature infants through active treatment of PS and non-invasive ventilation in the early period. However, From DOL 7 to DOL 14, the trend of decline in RIMP in the BPD group was slower than that in the non-BPD group. The pulmonary vasculature in infants who develop BPD is characterized by reduced arterial number, medial hypertrophy, and distal muscularization of small peripheral arteries, and abnormal vasoreactivity [27, 28]. These changes lead to elevated pulmonary resistance and the limited decline of RIMP. In addition, infants subsequently diagnosed with BPD had a higher RIMP at DOL14 compared than did the non-BPD group, suggesting that the PVR of the infants with BPD was also higher during this period.

Our results reveal that TAPSE in the two groups showed a trend of gradual increase with age increasing, which reflects that the heart function of premature infants tends to mature. TAPSE increased from DOL 1 to DOL 7 similarly in the two groups. From DOL7 to DOL14, TAPSE increased more slowly in the BPD group than in the non-BPD group, and TAPSE measured on DOL 14 in the BPD group was significantly lower than that in the non-BPD group, which could be due to the limited decline in PVR in the BPD group during this period, resulting in increased right ventricular afterload, and thus affecting the right cardiac function of the infants. Seo and Choi observed higher RIMP and lower TAPSE values in infants with symptomatic early pulmonary hypertension than in their counterparts. However, their association with the risk of BPD development was not clear [29]. Sehgal et al. observed higher RIMP and lower TAPSE values in infants with BPD than in those without BPD and an association with the duration of subsequent respiratory support [13]. Overall, these findings suggest that premature infants with BPD may demonstrate changes in PVR and pulmonary artery pressure at an early age, which could be evaluated effectively using TAPSE and RIMP values.

In this study, the BPD group had lower PLT count and higher MPV than did the non-BPD group on DOL 1, and logistic regression showed that the PLT and MPV were also independent risk factors for BPD, which was consistent with previous studies [16–19]. Lefrancais et al. have shown that the lung is a potential hematopoietic organ, involved in the biogenesis of platelets [30]. Therefore, pulmonary lesions and abnormal microvascular morphology may affect the production and release of platelets in the lung during the onset and progression of BPD. MPV can reflect platelet size, function, and activity. Larger MPV corresponds to greater enzyme and particle release, which may promote inflammation. Dani et al. and Cekmez et al. both reported high MPV in the first days of life as associated with an increased risk of BPD in preterm infants [31, 32]. Otherwise, we found that the PLT count and MPV values were not comparable in both groups on DOL 7 and DOL 14,which may be related to the good prognosis of most BPD infants admitted to our hospital.

We found that Echocardiographic indices of the right ventricular function combined with platelet parameters measured seem to be a promising tool for early identification of infants at risk of subsequent BPD development. However, this study has some limitations. Although the logistic regression analysis was used to adjust for potential confounders, the findings in the present analysis could not totally avoid bias caused by differences in GA and birth weight between the two groups. Secondly, for newborn protection, the first echocardiography for some premature infants may be completed within 1 to 3 days after birth. Thirdly, this study was retrospective, and the sample size was relatively small. In the future, large prospective studies are required to validate these findings.

## Conclusion

Echocardiographic indices of the right ventricular function combined with platelet parameters measured may help predict BPD in preterm infants.

## Data Availability

The raw dataset analyzed in the current study are available from the corresponding author on reasonable request.

## Abbreviations

AUC: Areas under the curve
BPD: Bronchopulmonary dysplasia
BPD-PH: BPD associated with pulmonary hypertension
BW: Birth weight
CI: Confidence intervals
DOL 7: Day 7 of life
DOL 14: Day 14 of life
GA: Gestational age
Hs-PDA: Hemodynamically significant patent ductus arterious
LVEI: Left ventricular eccentricity index
MPV: Mean platelet volume
NRDS: Neonatal respiratory distress syndrome
NICU: Neonatal intensive care unit
OR: Odds ratios
PH: Pulmonary hypertension
PVR: Pulmonary vascular resistance
PLT: Platelet count
PDW: Platelet distribution width
PCT: Plateletcrit
PFO: Patent foramen ovale
PMA: Postmenstrual age
RIMP: Right ventricular index of myocardial performance
ROC: Receiver operating characteristics curve
RVSP: Right ventricular systolic pressure
PASP: Pulmonary systolic pressure
TAPSE: Tricuspid annular plane systolic excursion
TRJV: Tricuspid regurgitation jet velocity
